# Optimization of Synthesis Parameters for Mesoporous Shell Formation on Magnetic Nanocores and Their Application as Nanocarriers for Docetaxel Cancer Drug

**DOI:** 10.3390/ijms140611496

**Published:** 2013-05-30

**Authors:** Ahmed Mohamed El-Toni, Mohamed Abbas Ibrahim, Joselito Puzon Labis, Aslam Khan, Mansour Alhoshan

**Affiliations:** 1 King Abdullah Institute for Nanotechnology, King Saud University, Riyadh 11451, Saudi Arabia; E-Mails: jlabis@ksu.edu.sa (J.P.L.); aslamkhan@ksu.edu.sa (A.K.); mhoshan@ksu.edu.sa (M.A.-H.); 2 Central Metallurgical Research and Development Institute (CMRDI), P.O. Box 87, Helwan, Cairo 11421, Egypt; 3 Department of Pharmaceutics, Faculty of Pharmacy, King Saud University, Riyadh 11451, Saudi Arabia; E-Mail: mhamoudah@ksu.edu.sa; 4 Department of Pharmaceutics and Industrial Pharmacy, Faculty of Pharmacy, Al-Azhar University, Assiut 71111, Egypt; 5 Department of Chemical Engineering, King Saud University, Riyadh 11421, Saudi Arabia

**Keywords:** mesoporous shell, Fe_3_O_4_, drug control release, core-shell, anionic surfactant

## Abstract

In this work, Fe_3_O_4_@SiO_2_ nanoparticles were coated with mesoporous silica shell by S^−^N^+^I^−^ pathway by using anionic surfactant (S^−^) and co-structure directing agent (N^+^). The role of co-structure directing agent (CSDA) is to assist the electrostatic interaction between negatively charged silica layers and the negatively charged surfactant molecules. Prior to the mesoporous shell formation step, magnetic cores were coated with a dense silica layer to prevent iron oxide cores from leaching into the mother system under any acidic circumstances. However, it was found that both dense and mesoporous coating parameters affect the textural properties of the produced mesoporous silica shell (*i.e.*, surface area, pore volume and shell thickness). The synthesized Fe_3_O_4_@SiO_2_@m-SiO_2_ (MCMSS) nanoparticles have been characterized by low-angle X-ray diffraction, transmission electron microscopy (TEM), and N_2_ adsorption-desorption analysis, and magnetic properties. The synthesized particles had dense and mesoporous silica shells of 8–37 nm and 26–50 nm, respectively. Furthermore, MCMSS possessed surface area of *ca.* 259–621 m^2^·g^−1^, and pore volume of *ca.* 0.216–0.443 cc·g^−1^. MCMSS showed docetaxcel cancer drug storage capacity of 25–33 *w*/*w*% and possessed control release from their mesochannels which suggest them as proper nanocarriers for docetaxcel molecules.

## 1. Introduction

Controlled drug release and stimulated drug targeting could be considered two of the most important and attractive research fields. The drugs carriers are critical for controlling drug storage volume and its release rate. In recent decades, significant advances in the development of drug carriers have been conducted [1].

A wide number of organic systems have been investigated as candidates for drug carriers in drug delivery systems, such as micelles [2], lipsomes [3], and polymers [4]. However, they suffer from various limitations, such as poor thermal and chemical stability, and rapid elimination by the immune system. In contrast, amorphous silica materials offer a biocompatible, stable and ‘stealthy’ alternative. Amorphous mesoporous silica materials have a non-toxic nature, very high specific surface, ordered mesoporous structure, tunable pore sizes and volumes area with abundant Si–OH bonds on the pore surface, and are mechanically stable, which makes them promising candidates for use as carriers in drug delivery systems [5–7].

By combining the advantages of mesoporous silica and magnetic particles to fabricate a nanocomposite with high surface area and magnetic separability, a novel adsorbent material and targeted drug delivery matrix, which carries the drug directly to a specific organ or location in the body under an external magnetic field will be available. Iron oxide, cobalt, or other magnetic nanoclusters have been incorporated into the pores of mesoporous silica, such as MCM-41 and SBA-15 [8]. However, in these cases, the pores were obviously clogged by the introduced particles. Meanwhile, the intensity of magnetism of the loaded silica mesoporous composite was weak due to its low loading level of magnetic clusters. Very recently, Lu *et al.* [9] reported a new method for fabricating magnetic mesoporous silica with open pores by grafting cobalt nanoparticles on the outer surface of the SBA-15 particles. Wu *et al.* [10] also synthesized magnetic composite with mesoporous silica being coated on Fe_3_O_4_ cores. However, the core-shell particles showed a wide particle size distribution from hundreds of nanometers to several microns. In addition, the particle sizes reported in all of these studies mentioned above are too large to be used for drug delivery, in which a particle size range between 50 and 300 nm is strictly demanded. Above 300 nm, a significant proportion of particles will be trapped in the lungs and liver, while too small particle size will cause the magnetic forces of these tiny particles to be too weak to work for separation or drug delivery. Zhao *et al.* [11] successfully fabricated magnetic core/mesoporous silica shell nanospheres, with a uniform particle diameter of *ca.* 270 nm, by sol-gel polymerization of tetraethoxysilane (TEOS), and n-octadecyltrimethoxysilane as porogen to generate the porosity. The inner magnetic Fe_3_O_4_/Fe core endues the nanoparticles with magnetic properties. The outer mesoporous silica shell shows moderate surface area and pore volume where drug molecules were stored in the channels of the nanospheres.

However, most of the research directed to synthesize solid core-mesoporous shell architectures was based only on a few reports [11–13] that revealed a solid core with disordered mesopores using n-octadecyltrimethoxysiliane (C18-TMS), whereas a few reports [14–16] have achieved a solid core with ordered mesopores using cetyltrimethylammonium bromide (CTAB) as a structure-directing agent through S^+^I^−^ pathway. Implementation of different kinds of surfactant, rather than a cationic one, could pose a significant impact on the textural properties of mesoporous silica shell.

Recently, Che and Tatsumi reported the synthesis of highly ordered anionic surfactant templated mesoporous silica (AMS) materials with anionic surfactants and co-structuredirecting agent (CSDA) through a new S^−^N^+^I^−^ pathway, where S stands for surfactant, N stands for CSDA, and I stands for inorganic precursors [17]. In this pathway, aminosilane (e.g., 3-aminopropyltrimethoxysilane) or quaternized aminosilane (e.g., N-trimethoxylsilylpropyl-N,N,N-tributylammonium) were used as CSDA. During the self-assembly process, the positively charged amine or ammonium sites of CSDA interact electrostatically with the templating anionic surfactant micelles and the alkoxysilane sites of CSDA co-condense with the inorganic precursors. This new pathway has been proven a successful way to produce a series of novel mesostructured phases, such as lamellar, hexagonal, cubic, and disordered mesostructures [18,19] as well as well-defined morphologies [20–22]. In addition, mesoporous silica synthesized through the S^−^N^+^I^−^ pathway would be beneficial to the preparation of surface amino-functionalized mesoporous silica after simple removal of the anionic surfactant by an acid-extraction [23]. To the best of our knowledge, the optimization of S^−^N^+^I^−^ pathway parameters for fabrication of mesoporous shell on magnetic cores have not reported yet. In this work, mesoporous silica shell formation onto super magnetic Fe_3_O_4_@SiO_2_ nanoparticles (MCMSS) was optimized by using anionic surfactant in a two-pot synthesis route. The impact of both dense and mesoporous coating parameters on surface area, pore volume and shell thickness was studied. MCMSSs have been tested as nanocarriers for docetaxcel cancer drugs where they have shown high storage capacity together with slow release rates.

## 2. Results and Discussion

### 2.1. Synthesis of Fe_3_O_4_ Nanoparticles

**Figure 1:**
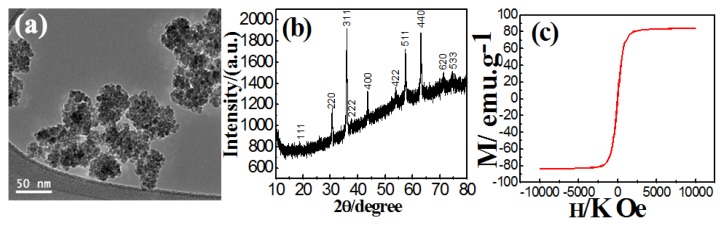
(**a**) Transmission electron microscopy (TEM) image; (**b**) Wide-angle X-ray diffraction; (**c**) Room-temperature magnetization curve for magnetite nanoparticles prepared by tri-sodium citrate.

Magnetite nanoparticles with a mean diameter of 40 nm were synthesized by solvent-thermal reaction using tri-sodium citrate as shown in Figure 1a. Magnetite nanoparticles showed a X-ray pattern that can be easily indexed to Fe_3_O_4_ (JCPDS Card No. 75-1609), Figure 1b. Absence of impurity peak suggested the successful formation of high purity magnetite product under current experimental conditions.

The magnetic properties of the magnetite particles indicate that the particles have no remanence or coercivity at 300 K, and its magnetization saturation value is (Ms) 83 emu/g as shown in Figure 1c.

### 2.2. Dense Silica Coating of Magnetic Nanoparticles

To fabricate mesoporous silica shell onto Fe_3_O_4_ nanoparticle by anionic surfactant, first Fe_3_O_4_ should be coated with a dense silica layer of desired thickness in order to protect the iron oxide core from leaching into the mother system under any acidic circumstances. Silica coating parameters are the tool to tune the thickness of this silica layer and to minimize the Fe_3_O_4_ particle agglomeration. In the coming part, we will investigate the effect of different coating parameters as ethanol concentration; ammonia concentration and Fe_3_O_4_ particle type (see Supplementary Information).

#### 2.2.1. Effect of Ethanol Concentration

**Figure 2:**
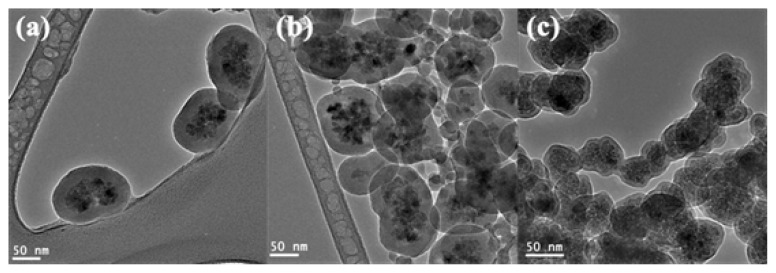
TEM images of silica coated Fe_3_O_4_ stabilized by tri-sodium citrate and prepared by (**a**) 0.8; (**b**) 0.84 and (**c**) 0.87 g/mL ethanol and 0.03 g/mL ammonia.

The addition of ethanol during the silica coating process is crucial since ethanol acts as a solvent for TEOS. Increasing ethanol concentration from 0.8 to 0.84 and 0.87 g/mL resulted in the decrease of silica layer thickness from 25 to 21 and 10 nm, respectively as shown in Figure 2. Moreover, silica coated Fe_3_O_4_ nanoparticles tended to agglomerate with increasing ethanol concentrations. The decrease of silica layer thickness with increasing ethanol content can be attributed to the dilution effect of ethanol which is retarding the TEOS hydrolysis and condensation reactions. Therefore, silica particles nucleation was initiated at a higher super-saturation, resulting in thinner silica layer [24]. The agglomeration of silica coated magnetic nanoparticles at 0.87 g/mL ethanol concentration can be attributed to poor dispersity of Fe_3_O_4_ due to increase of ethanol to water ratio, leading to the formation of irregular silica-coated aggregates of Fe_3_O_4_ nanoparticles [25].

#### 2.2.2. Effect of Ammonia Concentration

**Figure 3:**
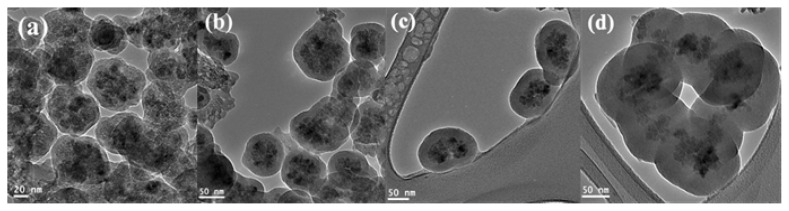
TEM images of silica coated Fe_3_O_4_ stabilized by tri-sodium citrate and prepared by (**a**) 0.008; (**b**) 0.016; (**c**) 0.03 and (**d**) 0.06 g/mL ammonia and 0.8 g/mL ethanol.

It is known that ammonia catalyzes the hydrolysis and condensation of the alkoxysilanes. Thus, it is expected that silica layer thickness will increase with increasing ammonia concentrations. Non-porous silica thickness was 8, 14, 25, and 37 nm at 0.008, 0.016, 0.03, and 0.06 g/mL ammonia concentrations, respectively, as shown in Table 1 and Figure 3. In addition, the particle agglomeration increased as ammonia concentration is prompted. It seems that the ammonia concentration threshold at which the silica coated Fe_3_O_4_ particles are highly agglomerated is 0.06 g/mL. Gradual increase of ammonia concentration leads to the increases of ionic strength of the reaction medium which catalyzes the hydrolysis and condensation of the TEOS and causes the increase in the shell thickness [26]. Exceeding 0.03 g/mL ammonia causes a serious agglomeration to take places due to very rapid hydrolysis and condensation.

### 2.3. Mesoporous Shell Formation on Silica Coated Magnetic Nanoparticles

To construct mesoporous silica shell for drug entrapment and controlled release, residual hydroxyl ions from dense silica coating, together with co-structure directing surfactant and TEOS, will be used for shell formation, and the factors influencing the mesoporous shell formation will be studied. These factors include some parameters of dense silica coating step and some other parameters of mesoporous shell formation. The dense silica coating factors that will affect the mesoporous shell formation are ethanol and ammonia concentrations. On the other hand, in the mesoporous shell formation step, the factor will be surfactant concentration.

#### 2.3.1. Dense Silica Coating Step Parameters Influencing Mesoporous Shell Formation

##### 2.3.1.1. Effect of Ethanol

**Figure 4:**
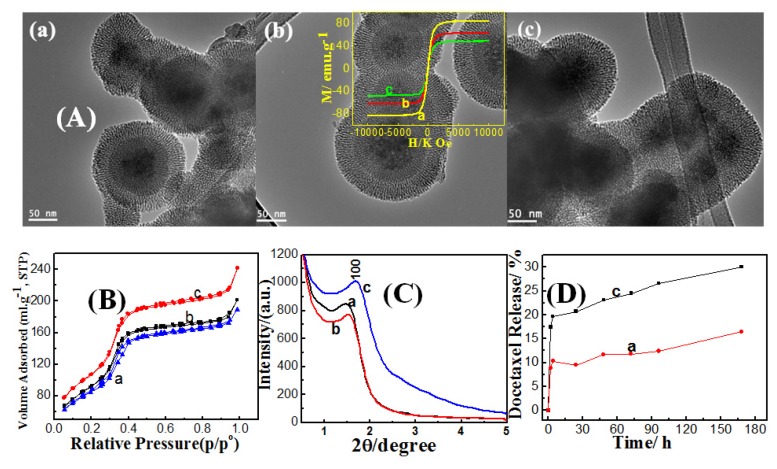
(**A**) TEM images; (**B**) N_2_ adsorption/desorption isotherms; (**C**) Low-angle X-ray diffraction for magnetic core-mesoporous silica shell prepared with different amounts of ethanol added during dense coating step (**a**) 0.8, (**b**) 0.84 and (**c**) 0.87 g/mL and (**D**) *In vitro* release study of docetaxel loaded at magnetic core-mesoporous silica shell prepared with different amounts of ethanol added during dense coating step (**a**) 0.8, and (**c**) 0.87 g/mL. Room-temperature magnetization curve for (**a**) magnetite nanoparticles, (**b**) dense silica coated magnetite nanoparticles (**c**) magnetic core-mesoporous silica shell (MCMSS) (inset).

As ethanol concentrations have affected the dense silica shell thickness, they also have a significant impact on mesoporous shell formation despite that the ethanol was not existent during this step. Mesoporous shell thickness had increased from 27, 36, to 43 nm for dense silica coated Fe_3_O_4_ samples prepared with ethanol concentration of 0.8, 0.84, to 0.87 g/mL, as shown in the TEM images (Figure 4A). It can be observed that at low ethanol content a clear core-shell structure could be identified. The dark Fe_3_O_4_ core was coated, firstly, with dense silica layer, and secondly, with mesoporous silica shell. It was also noticed that the dense and mesoporous layers can be distinguished at low ethanol content which is not possible at higher content. We cannot differentiate between the dense and mesoporous layers because the dense layer had converted to a porous one by attack of OH^−^ and adsorption of surfactant molecules, as described in the synthesis of double mesoporous core-shell silica spheres [27]. This concept can be also supported by high pore volumes of sample, prepared with high ethanol content, compared to lower ethanol content samples as shown in Table 1. At low ethanol content, the reactants can react faster and produce thicker silica layer. On the other hand, increasing the ethanol content diluted the system such that it reduced the thickness of dense silica layer. In this regard, thicker silica layer was less prone to the dissolution of siloxane bonds and the mesopores were not formed within dense silica shell. On the other hand, thinner silica shell became more susceptible to attack by OH^−^ and siloxane bonds break, which in turn resulted in mesopores formation. Consequently, the mesoporosity degree, expressed by pore volume, is expected to increase with increased ethanol content. However, the dense silica coated magnetite and magnetic core-mesoporous silica shell (MCMSS) samples showed a lower magnetization saturation values compared to non-coated sample as shown in the inset of Figure 4Ab.

Nitrogen adsorption/desorption isotherms measured at 77 K for magnetic core-mesoporous shell silica samples with different amounts of added ethanol during the dense coating step are shown in Figure 4B. The isotherms exhibited the type IV curves, which are characteristic of uniform mesoporous materials. The absence of the hysteresis loop is assigned to mesoporous matrices with cylindrical pores open at both ends [28]. The textural properties, BET surface area and total pore volume, of MCMSS were promoted with increasing ethanol content as described in Table 1. The conversion of first silica layer, from a completely dense to a mesoporous character, can be also confirmed from the fact that the total pore volume had significant increment, with increasing ethanol content, and this is consistent with the above mentioned explanation. On the other hand, the pore size distribution was narrow, with a peak centered at 3.6 nm; indicating uniform sizes of the mesopores (see Figure S2 Supplementary Information).

Low-angle X-ray diffraction spectra of the magnetic core-mesoporous shell silica samples with different amounts of ethanol added during dense coating step are shown in Figure 4C. Diffraction spectra show the appearance of single peak that can be assigned to the (100) diffraction peak, which can be indexed to a 2D hexagonal structure that suggest some sort of ordering in the mesoporous shell. The disappearance of other hexagonal peaks, (110) or (200), can be attributed to a certain extent to the distortion from a perfect 2D hexagonal mesostructure, due to the packing of the radially oriented mesopores in the spherical shell [29]. The MCMSS sample at 0.87 g/mL showed highest peak intensity compared to other samples, which suggests that the formed mesoporous shell had better ordering, and besides the shell possessed the most thickness.

*In-vitro* docetaxcel release for MCMSS samples, with different amounts of ethanol added during the dense-coating step, are shown in Figure 4D. The encapsulation efficiency of docetaxcel into MCMSS samples prepared with 0.87 and 0.8 g/mL ethanol is *ca.* 27.54, 33.8 wt%, respectively. Docetaxel have burst releases from the sample prepared by high ethanol content that shows superior surface area and pore volume; however the release rate becomes slower over a long release time. The sharp initial burst could be due to the rapid leaching of free drug molecules from the pore entrances [30]. MCMSS-1, having superior silica shell thickness and total pore volume, possessed lower deocetaxel storage capacity and faster release, compared with MCMSS-3 with lower textural properties. Such unexpected behavior of the MCMSS-1 sample can be attributed to the adsorption of some portion of docetaxcel molecules having taken place on the outer surface of the mesoporous silica shell, even after acid washing, rather than inside their inner mesochannels. On the other hand, MCMSS-3 showed slower release, which suggests that most of the docetaxcel molecules were trapped inside the inner cavity of mesochannels.

##### 2.3.1.2. Effect of Ammonia

**Figure 5:**
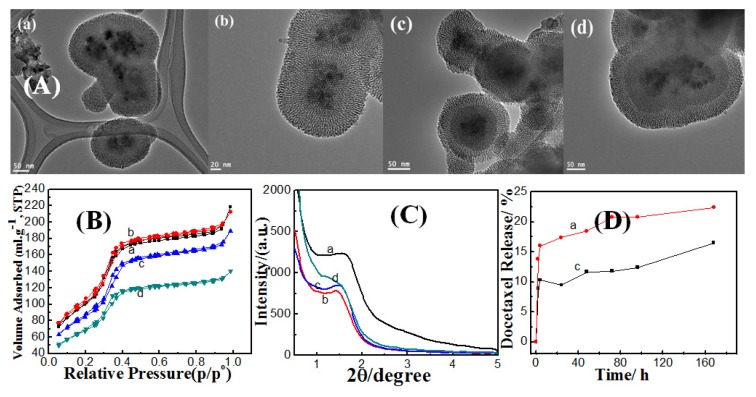
(**A**) TEM images; (**B**) N_2_ adsorption/desorption isotherms; (**C**) Low-angle X-ray diffraction for magnetic core-mesoporous silica shell prepared at different ammonia concentrations, added in the dense coating step of (**a**) 0.008, (**b**) 0.016, (**c**) 0.03 and (**d**) 0.06 g/mL and (**D**) *In-vitro* release study of docetaxel loaded at magnetic core-mesoporous silica shell, prepared at different ammonia concentrations added in the dense coating step of (**a**) 0.008, and (**c**) 0.03 g/mL.

Residual hydroxyl ions remaining from dense silica coating were expected to catalyze hydrolysis and condensation of TEOS during mesoporous shell formation. As shown in Figure 5A, magnetic core-mesoporous silica shell samples (MCMSS) underwent particle agglomeration, regardless of the concentration levels of ammonia that were being used. Mesoporous silica shell thickness was retarded from 50 nm to 44, 27 and finally 26 nm by increasing the ammonia concentration added during dense the coating step from 0.008 to 0.016, 0.03 and 0.06 g/mL, respectively. It can be noticed also that at very low ammonia concentration, 0.008 g/mL, the dense and mesoporous silica layers cannot be distinguished. With increasing ammonia concentration, the mesoporous shell thickness was promoted accordingly with a clear distinction between the dense and mesoporous layers. The differentiation of mesoporous and dense silica layers can be attributed to a protective silica layer that formed around magnetic cores, which become denser with increasing ammonia content and imparted a porous character at low ammonia concentration, as we have previously described in DMCSS. At low concentrations of ammonia (0.008–0.016 g/mL), thin silica shells were formed and the residual hydroxyl ions slightly induced pore formation at these thin and dense silica shells, as showed in Figure 5Aa,b. Increasing ammonia concentration to 0.03 and 0.06 g/mL resulted in thicker silica shells of 25 and 37 nm, respectively. However, at such thickness, those shells seemed to be quite dense [31], which hinders the diffusion of OH^−^ ions. Despite the residual hydroxyl ions concentrations being considerably high, it could not cause mesopores formation within the dense shell. This behavior can be described as size-dependent etching, consistent with the findings of Park *et al.* [32].

X-ray diffraction patterns (Figure 5C) exhibit obvious diffraction peak at about 1.7° (2θ), corresponding to the (100) diffraction peak characteristic for hexagonal mesophase at all ammonia concentrations. However, the broadening (100) peak at 0.008 and 0.06 g/mL ammonia can be attributed to the loss of pore ordering. The loss of diffraction intensity may be due to the absence of mesopores within the dense silica shell.

The textural properties, surface area and total pore volume, of MCMSS were suppressed gradually as the ammonia concentration level was raised up to 0.06 g/mL. This behavior can be attributed to that at ammonia concentration of 0.008 g/mL, and during the mesoporous shell formation step, the dense silica layer imparted mesoporous character. Therefore, this sample possessed the superior BET and total pore volume values. Further increases of ammonia concentration from 0.016 to 0.06 g/mL made the silica protective layer gradually dense and become more resistive for conversion to mesoporous status. However, docetaxel release rates are more associated with textural properties of magnetic core-mesoporous shell samples. Samples prepared at low ammonia content (having high surface and pore volume) possessed more burst release than samples with higher ammonia content. Andersson *et al.* [33] have reported that the density of surface silanol groups in the pore walls are thought to have an impact on the drug loading efficiency, since the drugs interact via hydrogen bonds with surface silanol groups located inside the pores. Our results are consistent with the findings of Andersson *et al.,* since the samples prepared with high ammonia concentration, 0.03 g/mL, were expected to have higher silanol density within mesoporous shell walls, and consequently, can load more docetaxel molecules.

#### 2.3.2. Mesoporous Shell Formation Parameters

##### Effect of Surfactant

**Figure 6:**
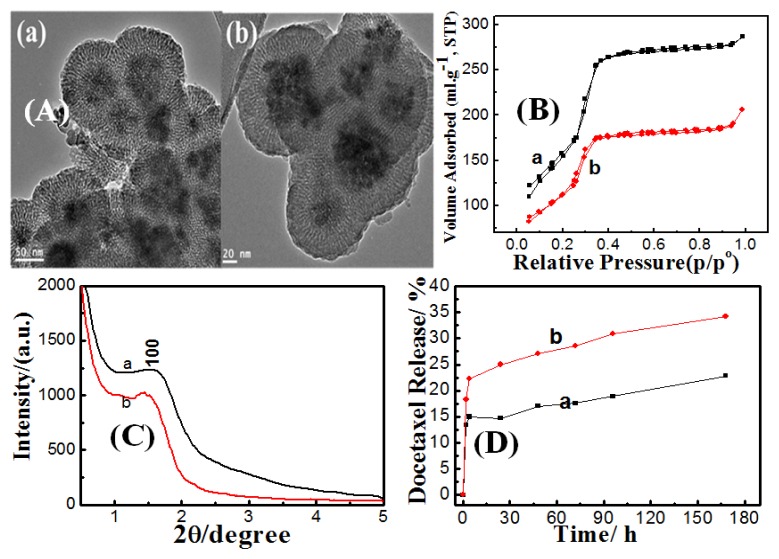
(**A**) TEM images; (**B**) N_2_ adsorption/desorption isotherms; (**C**) Low-angle X-ray diffraction for magnetic core-mesoporous silica shell prepared at different surfactant concentrations of (**a**) 5 and (**b**) 1 mmol, and (**D**) *In-vitro* release study of docetaxel loaded at magnetic core-mesoporous silica shell prepared at different surfactant concentrations of (**a**) 5 and (**b**) 1 mmol.

Tuning surfactant concentration did not affect the formation of mesoporous shell formation as shown in TEM images in Figure 6A. Both samples underwent particle agglomeration and the silica shell thicknesses were estimated to be 37 and 40 nm at 1 and 5 mmol surfactant concentrations, respectively. However, at a low surfactant concentration of 1 mmol, separate mesoporous silica particles were formed as a by-product, as shown in Figure S3. This separate mesoporous silica by-product caused the increase of BET surface area and total pore volume, as shown in Figure 6B. BET surface area and pore volume at a 1 mmol surfactant concentration were 621.79 m^2^·g^−1^ and 0.443 cc·g^−1^, respectively. When increasing the surfactant concentration to 5 mmol, both surface area and porosity was reduced significantly to 470.5 m^2^·g^−1^ and 0.318 cc·g^−1^, respectively. Such significant reduction in the textural properties of MCMSS nanoparticles can be attributed to the disappearance of separate mesoporous silica nanoparticles, which are characterized by superior textural properties. This is reported by Jin *et al.* [34] who had indicated that the generation of separate mesoporous silica demands very rigorous ionization degree (for HCl/C16-L-Ala) of the amphiphilic molecule, which controls the molecular packing to form helical propeller-like micelle. The increase of acid/surfactant molar ratio increased the ionization degree of the anionic amphiphilic molecules and thus increased its electrostatic interaction with the quaternary ammonium group of CSDA. The strong interaction between the surfactant head group and quaternary ammonium group of CSDA will cause the formation of separate mesoporous silica [34].

At low surfactant concentration, acid/surfactant molar ratio was quite high in order to achieve the conditions for obtaining separate mesoporous silica (acid was added to hydrolysize surfactant molecules). However, separate mesoporous silica was not the predominant one, but also some part of the surfactant molecules were adsorbed onto silica coated Fe_3_O_4_ nanoparticles to form core-mesoporous shell structure. Consequently, when silica hydrolysis begins to take place, after the addition of CSDA and TEOS, two separate mesophases were formed. The first phase was mesoporous silica shell surrounding Fe_3_O_4_@SiO_2_ and the second one was separate mesoporous silica. On the other hand, when surfactant molecules were increased to 5 mmol, as conducted in all previous experiments, the acid/surfactant molar ratio was enough to allow for the formation of only one type of micelles, one mesophase, which was adsorbed onto Fe_3_O_4_ nanoparticles. Therefore, the separate mesoporous silica nanoparticles disappeared in the production of this experiment. However, MCMSS samples prepared by 1 and 5 mmol surfactant concentrations have limited pore ordering, as indicated from the X-ray diffraction pattern shown in Figure 6C. In addition, changing surfactant concentrations did not result in a significant impact on the *in vitro* release of docetaxel molecules despite that the sample prepared at 1 mmol surfactant possessed a high surface area and pore volume due to the formation of separate mesoporous silica by-product. However, it seems that those separate mesoporous silica particles did not have affinity for the entrapment of docetaxel molecules.

## 3. Experimental Section

### 3.1. Synthesis

#### 3.1.1. Fe_3_O_4_ Synthesis

FeCl_3_·6H_2_O (1.35 g, 5 mmol) was dissolved in ethylene glycol (40 mL) to form a clear solution, followed by the addition of sodium acetate (NaAc) (3.6 g) or tri-sodium citrate (Na_3_Cit) (1.3 g) and polyethylene glycol (1.0 g). The mixture was stirred vigorously for 30 min and then sealed in a teflon lined stainless-steel autoclave (50 mL capacity). The autoclave was heated to and maintained at 190 °C for 18 h, and allowed to cool to room temperature. The black products were washed several times with ethanol and dried at 60 °C for 6 h.

#### 3.1.2. Dense Silica Coating

Magnetic cores were coated with dense silica layer to prevent iron oxide cores from leaching into the mother system under any acidic circumstances. Briefly, 0.10 g of Fe_3_O_4_ particles (~50 nm in diameter) were dispersed in the mixture of ethanol (30–50 mL), deionized water (2.6 mL) and concentrated ammonia aqueous solution (0.3–2.4 mL), followed by the addition of tetraethyl orthosilicate (TEOS, 0.03 g). After stirring at room temperature for 1.5 h, the silica coated Fe_3_O_4_ nanospheres were separated and washed with ethanol and water.

#### 3.1.3. Construction of Mesoporous Silica Shell by Anionic Surfactant

To form the mesoporous silica shell, silica coated magnetic nanoparticles were dispersed in 25 mL of H_2_O by ultrasonication for 10 min. For suppressing the agglomeration of silica cores, 1 g/L of polyvinylpyrrolidone, was added with continuous stirring for 60 min. Thereafter, 0.10 mL of 3-aminopropyltrimethoxysilane, 0.2933 g (1 mmol) of N-lauroylsarcosine sodium (acidified solution) and 1.5 mL of TEOS were added to the reaction mixture with subsequent stirring at 50 °C for 2 h. The final solid was recovered by centrifugation, washed with deionized water and dried in an oven at 60 °C for 12 h. The resulting molar ratio was:

$$\text{TEOS}:{\text{H}}_{2}\text{O}:\text{APMS}:\text{Sar}-\text{Na}:\text{HCl}:\text{PVP}=1.331.6:0.08:0.14:0.06:5\times 10^{-3}$$

### 3.2. Docetaxel Loading and Release Study

A 25 mg of magnetic core-mesoporous shell silica (MCMSS) sample was added into 25 mg/mL docetaxel ethanol solutions. The suspension was stirred for 2 h while the evaporation of ethanol was prevented. The MCMSS with drug loaded were separated by high-speed centrifugation and dried in a vacuum oven at 60 °C. 1.0 mL filtrate was extracted with a vial, diluted to 100 mL and then analyzed by UV/Vis spectroscopy at 230 nm for docetaxel, respectively. A calibration curve for docetaxel was created by plotting absorbance *versus* docetaxel concentrations between 0 and 200 mg/mL. Drug EE% was calculated using the following formula:

EE%=actual amount of drug in nanoparticles/theoretical amount of drug in nanoparticles×100

To ensure that the adsorbed docetaxel onto outer surface of magnetic core- mesoporous shell silica (MCMSS) nanoparticles was removed before performing the release experiments, MCMSS/docetaxcel nanoparticles were washed twice with 0.1 N HCl (since docetaxel is basic drug) to remove any uncapsulated drug. Thereafter, nanoparticles were collected by centrifugation and finally, were dried.

For release studies, 10 mg Dox/MCMSS samples were immersed separately in 10 mL volume of simulated body fluid (SBF) at 37 °C, at a stirring rate of 100 rpm. Then, 2.0 mL release medium was removed for analysis at given intervals with a syringe and the same volume of fresh release medium was injected. The extracted medium was diluted to a desired concentration with simulated body fluid and analyzed by UV/Vis spectroscopy as above.

### 3.3. Characterization

Transmission electron microscopy (TEM) images were obtained using a JEOL JSM-2100F electron microscope (Tokyo, Japan, Japan) operated at 200 kV. Powder X-ray diffraction (XRD) patterns were recorded on a PANalytical X’Pert PRO MPD (PANalytical, Lelyweg, Netherlands) with Ni-filtered Cu Kα radiation (45 kV, 40 mA). Nitrogen sorption isotherms were measured at 77 K with a Quantachrome NOVA 4200 analyzer (Quantachrome, Boynton Beach, Fla USA). Before taking measurements, the samples were degassed in a vacuum at 200 °C for at least 18 h. The Brunauer-Emmett-Teller (BET) method was utilized to calculate the specific surface areas using adsorption data at the relative pressure range from 0.02 to 0.20. By using the Barrett-Joyner-Halenda (BJH) model, the pore volumes and size distributions were derived from the adsorption branches of isotherms and the total pore volumes (Vt) were estimated from the adsorbed amount at a relative pressure P/P_0_ of 0.995. The UV/Vis absorbance spectra were measured with a Shimadzu UV-2550 UV-Vis Spectrophotometer (Shimadzu, Tokyo, Japan).

## 4. Conclusions

Fabrication of mesoporous silica shell on magnetic cores by anionic surfactant using S^−^N^+^I^−^ route was successfully optimized. The textural properties, BET and total pore volume, of magnetic core-mesoporous silica shell (MCMSS) were promoted with increasing ethanol content. On the other hand, the textural properties tended to decrease with increasing ammonia concentration. Exceeding ammonia concentration over 0.016 g/mL causes the silica protective layer to become denser and resist its conversion for mesoporous character. Tuning surfactant concentration did not affect the formation of mesoporous shell, but separate mesoporous silica particles were formed as a by-product. Changing the synthesis conditions affected the docetaxel encapsulation efficiency and release rate. The release behavior varied according to synthesis conditions and the textural properties of MCMSS nanoparticles.

## Supplementary Information



## Figures and Tables

**Table 1:** Impact of synthesis parameters of magnetic core-mesoporous shell silica spheres on their textural properties and docetaxcel entrapment efficiencies.

Sample code	NH_4_OH-1st a g/mL	EtOH g/mL	Surfactant mmol	HCl g/mL	Shell b 1st 2nd/nm		BET cm^2^/g	Vp c Cc/g	Rp d /nm	Drug EE e /%
MCMSS1	0.03	0.87	5	0.16	10	43	411.09	0.373	3.6	27.54
MCMSS2	0.03	0.84	5	0.16	21	36	352.9	0.311	3.8	-
MCMSS3	0.03	0.8	5	0.16	25	27	319.81	0.292	3.6	33.81
MCMSS4	0.06	0.8	5	0.16	37	26	259.62	0.216	3.6	-
MCMSS5	0.016	0.8	5	0.16	14	44	389.32	0.328	3.8	-
MCMSS6	0.008	0.8	1	0.16	8	37	621.79	0.443	3.6	31.03
MCMSS7	0.008	0.8	5	0.16	8	50	386.94	0.338	3.8	25.67

a NH_4_OH-1st: NH_4_OH concentration in first step;

b first and second shell thickness;

c Vp: total pore volume;

d Rp: pore diameter;

e Drug EE: Drug Encapsulation Efficiency.
