# Supplementary material for: Optimization of Synthesis Parameters for Mesoporous Shell Formation on Magnetic Nanocores and Their Application as Nanocarriers for Docetaxel Cancer Drug
Source: Int J Mol Sci. 2013 May 30;14(6):11496–509. doi: 10.3390/ijms140611496 (PMC3709744; doi:10.3390/ijms140611496)

Supplementary Information

Effect of Fe_3O_4 Particle Type

The Fe_3O_4 nanoparticles prepared by different stabilizing agents, tri-sodium citrate and sodium acetate, are shown in Figure S1. Silica layer thickness was 26 and 25 nm for Fe_3O_4, stabilized by tri-sodium citrate and sodium acetate, respectively. However, silica coated Fe_3O_4 prepared by tri-sodium citrate were less agglomerated than silica coated Fe_3O_4 stabilized by sodium acetate. This suggested that tri-sodium is better in stabilizing Fe_3O_4, which can be attributed to tri-sodium citrate's possession of three carboxylate groups with strong coordination affinity to Fe III ions, which favors the attachment of citrate groups on the surface of the magnetite nanocrystals and prevents them from aggregation.

Figure S1. TEM images of silica coated Fe_3O_4 prepared by (**a**) tri-sodium citrate and (**b**) sodium acetate at 0.8 g/mL ethanol and 0.03 g/mL ammonia.

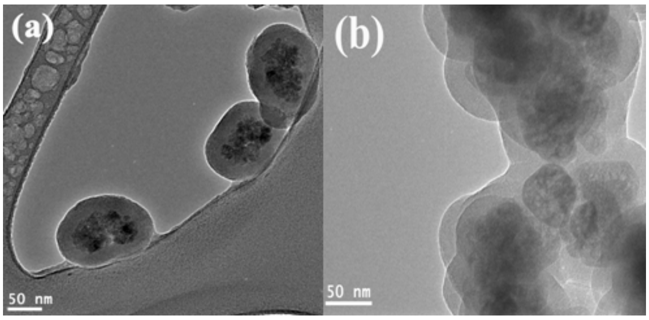

Figure S2. Pore-size distribution from the adsorption branch for magnetic core-mesoporous silica shell prepared at different ethanol concentrations of (**a**) 0.8; (**b**) 0.84 and (**c**) 0.87 g/mL.

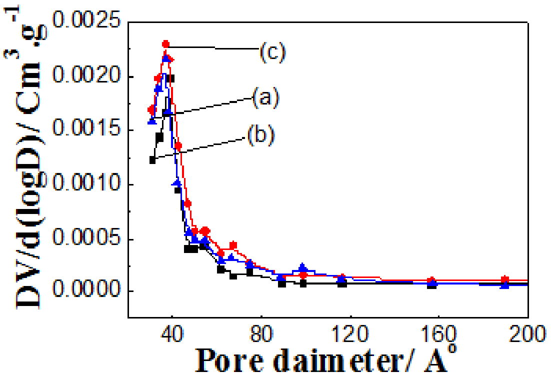

Figure S3. TEM image of mixture of chiral mesoporous silica and silica coated Fe_3O_4 core-mesoporous silica shell prepared at surfactant concentrations of 1 mmol.

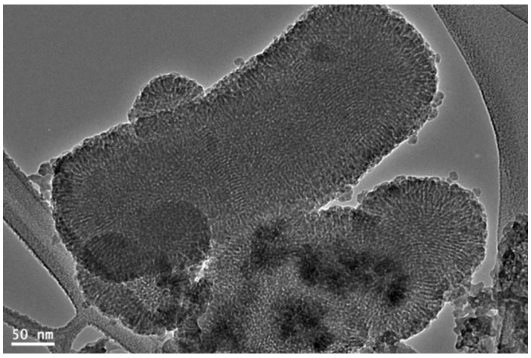

Supplement: Supplementary file 1 [file ijms-14-11496-s001.pdf]
